# Inhalation and Consumption

**Published:** 1857-10

**Authors:** 


### INHALATION AND CONSUMPTION.

MEDICATED INHALATION began to attract attention in New York about the close of 1854, in consequence of a certificate of its curative power given by the Mayor of Brooklyn, in reference to his wife, who is still an invalid. During the following year of 1855, and the beginning of 1856, newspaper aid was brought into requisition for the purpose of presenting this mode of treatment to the public. During that time, six of the leading daily papers are represented to us, on substantial authority, to have received twenty-one thousand dollars, from one practitioner of inhalation, for advertising its virtues. For days and weeks and months together, whole columns of the newspapers were devoted to the glorification of Inhalation, in separate advertisements and special editorials.

As evidence of its value in diseases of the throat and lungs, it was published, in February, 1856, that the deaths from *consumption* had diminished largely, and that this diminution was owing to the curative agency of Medicated Inhalation. It was further stated, that, by increased skill in its application, more favorable results might be confidently anticipated. And now that the increased skill, tact, and experience of two years are in full exercise, what are the results, as exhibited in the official reports of our courteous and indefatigable City Inspector, G. W. Morton, Esq.? During the *first half* of the last four years, the number of deaths from consumption in the City of New York is as follows:

| | 1854. | 1855. | 1856. | 1857. |
|---|---|---|---|---|
| Deaths by consumption, | 1,592 | 1,453 | 1,224 | 1,405 |
| Total deaths, | 11,940 | 11,563 | 9,507 | 10,834 |
| Total whole year's deaths, | 28,568 | 23,042 | 21,658 | 21,500 estimated. |

1st. By these tables, it will be seen that the diminution of deaths by consumption for 1854, '5, and '6, were *less* in proportion than the diminution of deaths from all causes.

2d. That instead of increased experience and skill in Medicated Inhalation operating to diminish the consumptive mortality still further, that mortality, for the first half of 1857, is largely greater than during the first half of 1856.

Nothing that we could say could more strongly exhibit the utter worthlessness of this treatment in the cure of consumptive disease. Our great regret is, that the newspaper press should have so hastily lent itself to the propagation of the imposition upon its patrons; and our mortification is, that so many young physicians, from all parts of the country, made such a disgraceful exhibition of their ignorance of their profession, as to write letters of inquiry as to whether it was an efficacious treatment or not. For had they known anything of medical history, they would have at once seen that it was an exploded theory, revived and rejected, time after time. Revived by ignorant or knavish men, and rejected by the educated of all nations, on precisely the same principle that a finished mechanician rejects the idea of perpetual motion, simply because, in the very nature of things, it is an impossibility.

